# A pilot clinical study of the therapeutic antibody against canine PD-1 for advanced spontaneous cancers in dogs

**DOI:** 10.1038/s41598-020-75533-4

**Published:** 2020-10-27

**Authors:** Masaya Igase, Yuki Nemoto, Kazuhito Itamoto, Kenji Tani, Munekazu Nakaichi, Masashi Sakurai, Yusuke Sakai, Shunsuke Noguchi, Masahiro Kato, Toshihiro Tsukui, Takuya Mizuno

**Affiliations:** 1 Laboratory of Molecular Diagnostics and Therapeutics, The United Graduate School of Veterinary Medicine, Yamaguchi University, 1677-1 Yoshida, Yamaguchi, Yamaguchi 753-8515 Japan; 2 Veterinary Teaching Hospital, Azabu University, Kanagawa, Japan; 3 Yamaguchi University Animal Medical Center, Yamaguchi University, Yamaguchi, Japan; 4 Laboratory of Veterinary Surgery, Joint Faculty of Veterinary Medicine, Yamaguchi University, Yamaguchi, Japan; 5 Laboratory of Veterinary Radiology, Joint Faculty of Veterinary Medicine, Yamaguchi University, Yamaguchi, Japan; 6 Laboratory of Veterinary Pathology, Joint Faculty of Veterinary Medicine, Yamaguchi University, Yamaguchi, Japan; 7 Laboratory of Veterinary Radiology, Graduate School of Life and Environmental Sciences, Osaka Prefecture University, Osaka, Japan; 8 Nippon Zenyaku Kogyo Co., Ltd., Koriyama, Fukushima Japan

**Keywords:** Cancer models, Cancer therapy

## Abstract

Inhibition of programmed death 1 (PD-1), expressed on activated T cells, can break through immune resistance and elicit durable responses in human melanoma as well as other types of cancers. Canine oral malignant melanoma is one of the most aggressive tumors bearing poor prognosis due to its high metastatic potency. However, there are few effective treatments for the advanced stages of melanoma in veterinary medicine. Only one previous study indicated the potential of the immune checkpoint inhibitor, anti-canine PD-L1 therapeutic antibody in dogs, and no anti-canine PD-1 therapeutic antibodies are currently available. Here, we developed two therapeutic antibodies, rat-dog chimeric and caninized anti-canine PD-1 monoclonal antibodies and evaluated in vitro functionality for these antibodies. Moreover, we conducted a pilot study to determine their safety profiles and clinical efficacy in spontaneously occurring canine cancers. In conclusion, the anti-canine PD-1 monoclonal antibody was relatively safe and effective in dogs with advanced oral malignant melanoma and other cancers. Thus, our study suggests that PD-1 blockade may be an attractive treatment option in canine cancers.

## Introduction

In human medicine, a variety of monoclonal antibodies are available for treatment of disease and excellent results have been achieved^1,2^. In veterinary medicine, however, there is only one caninized antibody lokivetmab in commercial use that targets canine interleukin-31 in dogs with atopic dermatitis^3,4^. Hence, a need exists for the development of effective monoclonal antibodies in veterinary applications.

Over the last decade, human cancer therapy has rapidly evolved due to solid evidence of the efficacy of immunotherapy. Monoclonal antibody-based immunotherapy has emerged as a measurable and attractive treatment option for a wide range of cancer patients. In particular, immune checkpoint blockades that target to cytotoxic T lymphocyte-associated antigen 4 (CTLA-4) and programmed cell death 1 (PD-1) have succeeded clinically in human melanoma patients and were approved by the FDA in 2011 and 2014, respectively^5^. Whereas CTLA-4 inhibits T cell responses by interacting with B7 proteins (CD80 and CD86)^6^, PD-1 interferes with T-cell receptor signaling by binding to two ligands, PD-L1 and PD-L2^7,8^. Since PD-1 signaling suppresses T-cell proliferation and cytokine production, PD-1 blockade reverses the PD-1-mediated inhibition of antitumor immunity. A fully humanized IgG4κ monoclonal antibody, nivolumab, and a humanized IgG4κ monoclonal antibody, pembrolizumab, have demonstrated high response rates with excellent durability in a variety of human cancers including melanoma, non-small cell lung carcinoma, and renal cancer^9,10^. Although some patients have developed intrinsic and acquired resistant phenotypes, immunotherapy with immune checkpoint inhibitors represents a breakthrough in cancer treatment^11^. Hence, we and other researchers suggest that canine spontaneous cancers may provide a powerful tool in translational studies between basic studies in mouse cancer models and human clinical studies^12^.

PD-L1 is expressed in many types of canine tumor tissues including malignant melanoma, mammary gland tumors, osteosarcoma, hemangiosarcoma, mast cell tumors, histiocytic sarcoma, renal cell carcinoma, lymphoma, and soft tissue sarcoma^13–17^. In addition, several monoclonal antibodies specific to canine PD-1/PD-L1 have been developed, and the functional activities of these have been confirmed in vitro and in vivo^16,18,19^. These studies show that stimulated lymphocytes induced the expression of PD-1 and PD-L1, and concluded that both anti-canine PD-1 and PD-L1 monoclonal antibodies activated the release of interferon-gamma (IFN-γ) from mitogen-activated lymphocytes or tumor infiltrating lymphocytes. Although previous studies revealed the potential of anti-PD-1/PD-L1 monoclonal antibodies as canine cancer treatment agents, only one pilot study of a chimeric antibody targeting canine PD-L1 showed the safety profile and clinical response in one out of seven oral malignant melanoma cases and one out of two undifferentiated sarcoma cases^20^.

Oral malignant melanoma (OMM) is one of the most aggressive tumors in dogs, and is characterized by rapid metastasis to other organs such as lung, lymph nodes, and liver. Moreover, dogs with spontaneously occurring melanoma may provide a preclinical model of human melanoma^21,22^. Surgical resection and radiation are first-line treatments for canine melanoma without metastasis^23,24^. Median overall survivals in 111 OMM cases with Stage I, II, III, and IV were 758 days, 278 days, 163 days, and 80 days, respectively^23^. These cases received orthovoltage and megavoltage radiation therapy, followed by adjunctive treatments of surgical resection and systemic chemotherapy. Although Stage IV OMM has poor prognosis, tumor response rate of chemotherapy against canine melanoma is limited and there is little evidence to indicate improved survival^24–26^. As a novel approach, an xenogeneic DNA vaccine encoding human tyrosinase (ONCEPT; Merial, Lyon, France) has been approved by the FDA as an adjunctive treatment for canine melanoma after surgical resection^27^. However, results of the efficacy of the DNA vaccine were inconsistent in several clinical studies^28–30^. Therefore, an effective systemic treatment against canine melanoma, especially in the advanced stage, is sorely needed.

In the present study, we prepared two therapeutic antibodies, rat-dog chimeric anti-canine PD-1 antibody (ch-4F12-E6) and caninized anti-canine PD-1 antibody (ca-4F12-E6), based on the anti-canine PD-1 antibody, 4F12-E6, from our previous study^19^. We evaluated in vitro reactivity and blocking capacity for these antibodies and reported the first evidence to our knowledge in pilot clinical study that anti-canine PD-1 monoclonal antibodies are relatively safety and effective in dogs with advanced OMM and other types of tumors. Thus, the present study demonstrated that PD-1 blockade therapy may be an attractive option in canine cancer therapy.

## Results

### Generation of chimeric and caninized anti-canine PD-1 monoclonal antibodies

**Figure 1:**
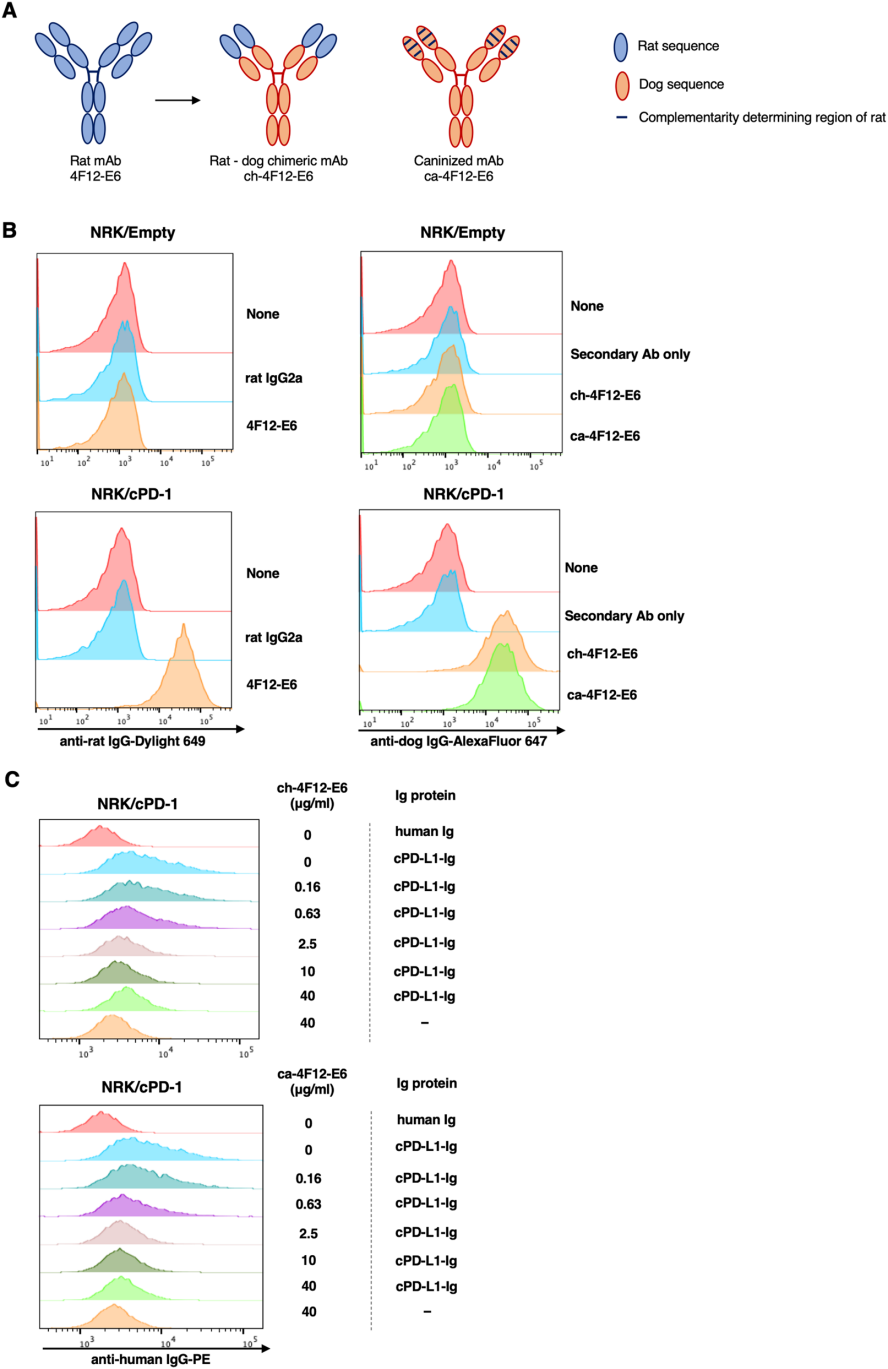
Establishment of chimeric and caninized anti-PD-1 monoclonal antibodies. (**A**) Schematic overview of rat monoclonal antibody (mAb) 4F12-E6, rat-dog chimeric mAb ch-4F12-E6, and caninized mAb ca-4F12-E6. (**B**) NRK cells expressing canine PD-1 (NRK/cPD-1) were stained with 40 μg/ml of rat IgG2a isotype control, 4F12-E6, ch-4F12-E6, or ca-4F12-E6 followed by incubation with secondary antibodies, anti-rat IgG-Dylight 649, or anti-dog IgG-AlexaFluor 647. As a control, cells were stained with anti-dog IgG-AlexaFluor 647 without primary antibody (indicated as Secondary Ab only). No antibodies bound to NRK cells transfected with empty vector. (**C**) NRK/cPD-1 cells were pre-incubated with dose-titrated monoclonal antibodies for ch-4F12-E6 or ca-4F12-E6, and, after washing, incubated with 40 μg/ml of cPD-L1-Ig or human Ig. Cells were then stained with secondary antibody anti-human IgG-PE. Representative data from at least two biological replicates are shown.

Previously, we developed and characterized a rat monoclonal antibody against canine PD-1 protein, referred to as 4F12-E6^19^. To reduce the immunogenicity of that antibody in dogs (as shown in Fig. 1A), we attempted to generate the rat-dog chimeric antibody (ch-4F12-E6) by fusing the heavy and light chain variable regions of 4F12-E6 with constant regions of dog IgG type A.

The rodent variable region itself could be a highly immunogenic protein, however, the chimeric antibody represents an improvement over the rodent antibody for use in dogs. Hence, we further reduced the rat-derived residues in the CDRs of heavy and light chain of variable regions by the PETization method (Fig. 1A) to obtain a caninized antibody, known as ca-4F12-E6, in which a total of 16% of the amino acids in the heavy and light chain variable regions were substituted.

In some cases, humanized antibodies had less binding affinity to target antigens compared with the original rodent antibodies because of deformation of the rodent’s CDRs. We first evaluated whether ch-4F12-E6 and ca-4F12-E6 could recognize canine PD-1 protein by flow cytometric analysis (Fig. 1B). These antibodies bound to canine PD-1 protein on NRK/cPD-1 cells, but did not recognize any proteins on NRK/Empty cells. This is consistent with results obtained for the 4F12-E6 original antibody, indicating that the conversion of variable regions did not influence on the potency of 4F12-E6.

### In vitro activity of chimeric and caninized anti-canine PD-1 monoclonal antibodies

To confirm that ch-4F12-E6 and ca-4F12-E6 exert their inhibitory effects on PD-1/PD-L1 binding, we constructed a soluble recombinant canine PD-L1 protein fused to human IgG Fc (cPD-L1-Ig). We used flow cytometric analysis to detect the binding capacity of cPD-L1-Ig to canine PD-1 expressed on NRK/cPD-1 cells. Moreover, we examined whether these monoclonal antibodies inhibited binding between PD-1 and PD-L1. Figure 1C showed that cPD-L1-Ig strongly bound to PD-1 protein on NRK/cPD-1 cells compared to human Ig as a control when there were no anti-canine PD-1 antibodies. In contrast, when NRK/cPD-1 cells were pre-treated with either ch-4F12-E6 or ca-4F12-E6 at various concentrations, the PD-1/PD-L1 binding was inhibited in a dose-dependent manner by those antibodies.

**Figure 2:**
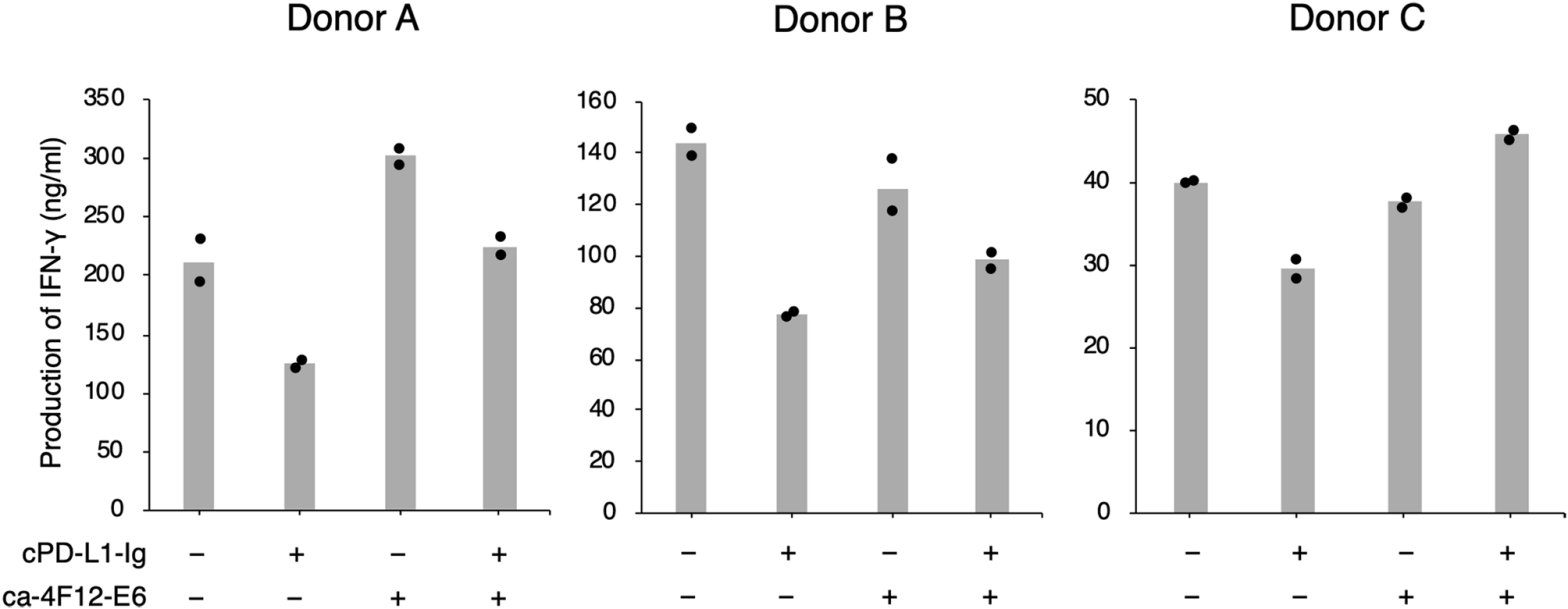
Suppression of interferon-gamma (IFN-γ) production by recombinant canine PD-L1 and restoration by caninized anti-PD-1 antibody. PBMCs were isolated from three healthy donors (donors A, B, and C) and stimulated with concanavalin A (5 μg/ml) in the presence of 10 μg/ml of dog IgG-A control or ca-4F12-E6. After 24 h, 5 μg/ml of human Ig or cPD-L1-Ig were added. The cells were cultured for 72 h, the supernatant was collected and the amount of canine IFN-γ in the supernatants measured in duplicate. Mean values of duplicates are displayed as a bar graph.

Since interferon (IFN)-γ secretion from activated lymphocytes is important in antitumor immunity, we tested the IFN-γ releasing assay in concanavalin A (ConA)-stimulated Peripheral blood mononuclear cells (PBMCs) isolated from three healthy dogs with cPD-L1-Ig and/or ca-4F12-E6 (Fig. 2). As controls, human Ig and dog IgG-A were used. Our previous study showed ConA induced the lymphocytic activation and PD-1 expression on PBMCs^19^. Figure 2 shows the variations in IFN-γ production in ConA-stimulated PBMCs between individual dogs. In all dogs (donors A–C), ConA-stimulated PBMCs cultured with cPD-L1-Ig demonstrated that production of IFN-γ was suppressed compared to control. This indicated that cPD-L1-Ig had a negative regulatory function on PBMC activation via PD-1/PD-L1 interaction. Whereas, adding of ca-4F12-E6 blocked the inhibitory effect of cPD-L1-Ig followed by enhancement of IFN-γ production in all dogs. In donor A, ca-4F12-E6 alone increased IFN-γ, this might be because anti-PD-1 antibody inhibited the auto-ligation of PD-1 on PBMCs via endogenous PD-L1 binding on adjacent PBMCs, a phenomenon that has been described previously. Even though the absolute values of IFN-γ augmented by treatments varied in each dog, these results suggested that anti-PD-1 antibody ca-4F12-E6 had functional activity for PD-1/PD-L1 binding.

### Baseline characteristics in dogs

Data for all enrolled dogs, including age, breed, sex, tumor type, staging, treatment history, number of doses of anti-PD-1 antibody, tumor response, and survival time, are shown in Table 1. A total of 30 dogs with a range of spontaneous canine cancers was enrolled in this study. Specifically, 21 cases represented OMM including four cases of Stage III and 17 cases of Stage IV. Other cases presented with mammary gland tumors (MGT), squamous cell carcinoma (SCC), renal carcinoma, lymphoma, sebaceous carcinoma, lung adenocarcinoma, and skin melanoma. Most cases had previously received conventional therapies including surgery, radiation, and systemic chemotherapy. However, three cases (Case No. 12, 22, and 30) had no prior therapies. Two cases (Case No. 5 and 15) had received a xenogeneic DNA vaccine encoding for human tyrosinase as an adjunctive treatment. PD-L1 expression status in each tumor tissues of this study was unknown, because commercial monoclonal antibody (rabbit anti-human PD-L1 monoclonal antibody (EPR1161, Abcam, Tokyo, Japan)), which can cross react with canine PD-L1 in immunohistochemical staining experiments as shown in our previous study^13^, was no longer available, and no alternative product is currently available.

**Table 1:** Baseline characteristics, treatment course, anti-drug antibody, and survival time in all enrolled dogs.

Case no	Breed	Age	Sex	Tumor type	Staging	Prior therapy	No. of PD-1 therapy	PD-1 Ab	Anti-drug Ab	Overall response at first time point	Overall response at subsequent time point	Progression-free survival (days)	Overall survival time (days)
1	Toy poodle	10Y	Female, spayed	Oral melanoma	Stage IV	Surgery, chemotherapy	11	ch-	–	SD	PD	168	353
2	Miniature Dachshund	14Y	Female, spayed	Oral melanoma	Stage IV	Radiation	6	ch-	–	PD	–	72	96
3	Beagle	10Y	Female, spayed	Oral melanoma	Stage IV	Radiation	17	ch-	–	PR	PD	161	257
4	Miniature Schnauzer	8Y	Male, castrated	Oral melanoma	Stage IV	Radiation, chemothrapy	6	ch-	–	PD	–	74	> 74
5	Miniature Dachshund	16Y	Male	Oral melanoma	Stage III	Sugery, radiation, chemotherapy, DNA vaccine	6	ch-	–	SD	–	> 77	185
6	Miniature Dachshund	14Y	Male	Oral melanoma	Stage IV	Surgery, radiation	6	ch-	–	PD	–	70	86
7	Yorkshire Terrier	13Y	Female, spayed	Oral melanoma	Stage IV	Surgery	10	ch-	–	PD	–	91	166
8	Toy poodle	10Y	Male	Oral melanoma	Stage IV	Surgery	3	ch-	NE	PD	–	14	56
9	Yorkshire Terrier	8Y	Male	Oral melanoma	Stage III	Surgery, radiation	6	ch-	–	PD	–	69	115
10	Chihuahua	14Y	Male, castrated	Oral melanoma	Stage IV	Surgery, radiation (kV)	3	ch-	–	PD	–	28	28
11	Sealyham Terrier	11Y	Female, spayed	MGT	Stage V	Surgery, chemotherapy	10	ch-	–	PD	–	74	260
12	Miniature Dachshund	12Y	Male	Oral melanoma	Stage IV	None	2	ch-	NE	NE	–	–	> 28
13	Toy poodle	16Y	Male, castrated	Oral melanoma	Stage IV	Radiation, chemothrapy	4	ch-	–	NE	–	56	56
14	Jack Russell Terrier	14Y	Female, spayed	SCC	Stage III	Radiation, Chemothrapy	4	ch-	–	PD	–	57	65
15	Chihuahua	13Y	Female, spayed	Oral melanoma	Stage IV	Surgery, radiation (kV), DNA vaccine	16	ch-	–	PR	PD	147	320
16	Shiba	15Y	Female, spayed	Oral melanoma	Stage III	Radiation	1	ch-	NE	NE	–	–	145
17	Miniature Schnauzer	8Y	Male	Skin melanoma	Stage III	Surgery	15	ch- ⟹ ca-	–	SD	SD	> 518	
18	Chihuahua	13Y	Female, spayed	SCC	Stage III	Surgery, radiation (kV)	7	ch-	–	NE	–	–	140
19	Miniature Dachshund	16Y	Male	Renal carcinoma	T2N0M1b	Radiation (kV)	13	ch- ⟹ ca-	–	PD	–	79	199
20	Miniature Dachshund	16Y	Male	Lymphoma	-	Chemotharapy	2	ch-	NE	NE	–	–	26
21	Miniature Dachshund	16Y	Male, castrated	Oral melanoma	Stage IV	Surgery	4	ch-	–	PD	–	28	51
22	Mix	9Y	Female	Sebaceous carcinoma and MGT	Stage III	None	5	ca-	–	PD	–	14	159
23	Miniature Schnauzer	13Y	Male, castrated	Oral melanoma	Stage IV	Surgery, chemotherapy	6	ca-	–	PD	–	42	307
24	Yorkshire Terrier	13Y	Male	Oral melanoma	Stage IV	Surgery, radiation (kV)	5	ca-	+	PD	–	70	95
25	French Bulldog	10Y	Male, castrated	Lung adenocarcinoma	T2 clinical stage	Surgery	5	ca-	–	PD	–	70	171
26	Miniature Schnauzer	12Y	Female, spayed	Skin melanoma	Stage IV	Surgery, chemotherapy	5	ca-	–	SD	–	84	275
27	Chihuahua	7Y	Male, castrated	Oral melanoma	Stage IV	Surgery, radiation (kV)	12	ca-	+	PR	PD	140	271
28	Pug	9Y	Male	Oral melanoma	Stage IV	Surgery, radiation	20	ca-	–	PD	PD	72	267 (ongoing)
29	Shizu	14Y	Male	Oral melanoma	Stage III	Surgery, radiation	10	ca-	–	NE	–	–	170
30	Labrador Retriever	12Y	Male	Oral melanoma	Stage IV	None	8	ca-	–	PR	PR	> 67	93 (ongoing)

### Treatment course

The dosing regimen and treatment cycle of anti-PD-1 antibodies were deduced from human clinical trials^31,32^. The chimeric anti-PD-1 antibody, ch-4F12-E6, and caninized ca-4F12-E6 were used in 19 and 9 cases, respectively. In two cases, ch-4F12-E6 was used in the first half-period, followed by caninized ca-4F12-E6. The median number of antibody administrations was 7.6 (range 1–20). Three cases (Case No. 12, 16, and 20) received ch-4F12-E6 treatment only once or twice due to rapid progression of the disease and owner’s request. No dose reduction due to any adverse events was performed in this study.

### Treatment-related toxicities

Treatment-related adverse events of any grade occurred in 19 out of 30 cases (63.3%). The most common adverse events were fever and gastrointestinal symptoms such as vomiting and diarrhea (Table 2). Adverse events greater than grade 3 were observed in 2 of 30 cases (6.7%). Case 14 had a grade 3 adverse event of fever. In Case 10, death was attributed to ch-4F12-E6 treatment. In this case, grade 5 inflammation marker C-reactive protein and pneumonitis presented the day after a three-dose treatment (Supplementary Fig. S1A,B). Despite treatment with glucocorticoids and intensive care, this dog died two days after symptoms started.

**Table 2:** Treatment-related adverse events in all enrolled dogs.

Case No	PD-1 Ab	Grading of adverse events							
		Fatigue	Anorexia	Fever	Vomiting	Diarrhoea	Pneumonitis (immune-related)	Tachypnoea	Tremor
Case1	ch-		1		1	1			
Case2	ch-	1			1	1			
Case3	ch-	1		1	1	1		1	
Case4	ch-								
Case5	ch-			1					
Case6	ch-			1	1	1			
Case7	ch-								
Case8	ch-				1				
Case9	ch-								
Case10	ch-	2		1			5	2	
Case11	ch-				1	1			
Case12	ch-								
Case13	ch-								1
Case14	ch-			3				1	1
Case15	ch-								
Case16	ch-	1	1		1	1			
Case17	ch- ⟹ ca-			2	1	1			
Case18	ch-			2					
Case19	ch- ⟹ ca-			1	1				
Case20	ch-			1					
Case21	ch-			2					
Case22	ca-								
Case23	ca-		1						
Case24	ca-					1			
Case25	ca-								
Case26	ca-								
Case27	ca-								
Case28	ca-								
Case29	ca-								
Case30	ca-							1	

Laboratory blood tests revealed various treatment-unrelated adverse events in most of the enrolled dogs, and severe liver damage related to administration of the anti-PD-1 antibody was observed in Case 1. This dog had elevated levels of alanine aminotransferase (ALT) and aspartate aminotransferase (AST) after having received eleven doses of ch-4F12-E6 (Supplementary Fig. S2A), and we decided to discontinue ch-4F12-E6 treatment on day 229 due to severe adverse events in this case. Ultrasonography, as well as a CT scan and biopsy were performed on day 235 to discriminate between melanoma metastasis and inflammation in the liver. The ultrasound image indicated a diffuse mixed echoic pattern as well as a nominal echo-poor area in the liver (Supplementary Fig. S2B), which was consistent with the CT scan. Biopsy specimens of the liver using a Tru-Cut needle documented a pericholangitis with mild mixed cell infiltration including plasma cells, lymphocytes, and neutrophils. However, tumor metastasis was not observed. We initiated treatment with a glucocorticoid as immune-related inflammation was suspected. As shown in Supplementary Fig. S2A, the levels of ALT and AST decreased after initiation of glucocorticoid treatment until day 251. However, levels of alkaline phosphatase (ALP) increased as a side effect of glucocorticoid treatment. We withdrew treatment on day 251 and began using the immunosuppressive drug mycophenolate mofetil instead of the glucocorticoid on day 283. As a result, liver enzymes decreased on day 343, however, multiple metastases of melanoma were observed in liver (Supplementary Fig. S2B). Case 1 died on day 353 and, during the autopsy, we found that mild lymphocytes had infiltrated peri-portal vein areas (Supplementary Fig. S2C) and melanoma cells had metastasized.

In contrast to ch-4F12-E6, as shown in Table 2, no treatment-related adverse events of more than grade 3 were observed in caninized ca-4F12-E6 treatment and the incidence of adverse events of any grade in ca-4F12-E6 (33.3%) was lower than that of ch-4F12-E6 (76.2%) despite the varied case size. These results suggest that caninized antibodies may be safer than chimeric antibodies because of their immunogenicity.

### Antitumor activity

The characteristics of tumor response to anti-PD-1 are shown in Table 1. In 24 of the 30 cases, tumor response was asssessed during treatment. The remaining cases were excluded because the tumor size was not measured accurately post-treatment or treatment with anti-PD-1 antibody was performed less than twice. In 17 cases, the target lesion was lung metastasis, and in 7 cases the target lesion was the primary mass or lymph nodes.

**Figure 3:**
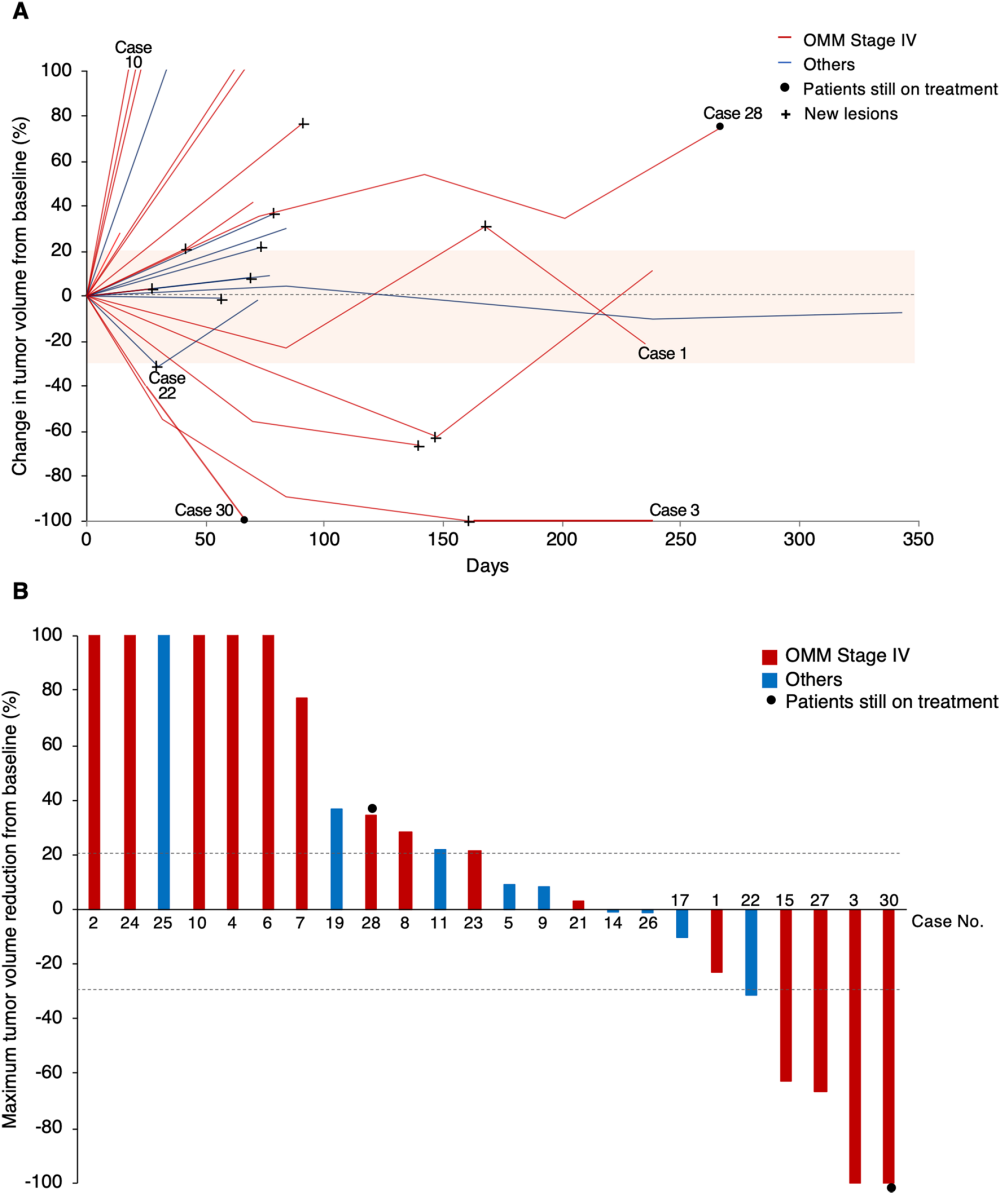
Clinical response to anti-PD-1 antibody in dogs with tumors. (**A**) A spider plot showing changes in tumor burden from baseline in 24 enrolled dogs. Red and blue lines indicate oral malignant melanoma (OMM) cases with Stage IV (n = 15) or others (n = 9), respectively. A cross (+) indicates the appearance of new lesions. Case 28 and Case 30 are still on treatment (indicated by a circle). End of line represents the termination of treatment. Even if a certain value is greater than 100%, the graph shows the value up to 100% as a matter of convenience. (**B**) A waterfall plot showing the maximum tumor volume reduction from baseline in Stage IV OMM cases (red bars) and others (blue bars) during treatment with the anti-PD-1 antibody. Dashed lines indicate values between − 30 and + 20%.

Figure 3A shows changes in tumor burden of the target lesion over time as distinguished between Stage IV OMM cases and others. Four cases in OMM cases with stage IV had objective responses with partial response (PR) at the first time point of evaluation (16.7%). The tumor burden of a target lesion in Case 30 was reduced completely, however non-target lesions remained. Case 22, which presented with oral sebaceous carcinoma and MGT, had tumor volume reduction of target lesions of less than − 30% on day 30 (before first assessment) accompanied by a new MGT lesion. Therefore, this case was defined as PD even though tumor burden was reduced (− 1.7%) from baseline at the end of first cycle of treatment. In addition, four of the 24 cases (16.7%) had stable disease and 16 cases (66.7%) had progressive disease. Case 28 and Case 30 had ongoing treatment after the final follow-up.

The waterfall plot (Fig. 3B) indicates the maximum reduction from baseline in tumor burden. Tumor shrinking or maintenance (maximum tumor volume reduction of less than 20%) occurred in 12 out of 24 cases (50.0%) including six Stage IV OMM cases and six others.

**Figure 4:**
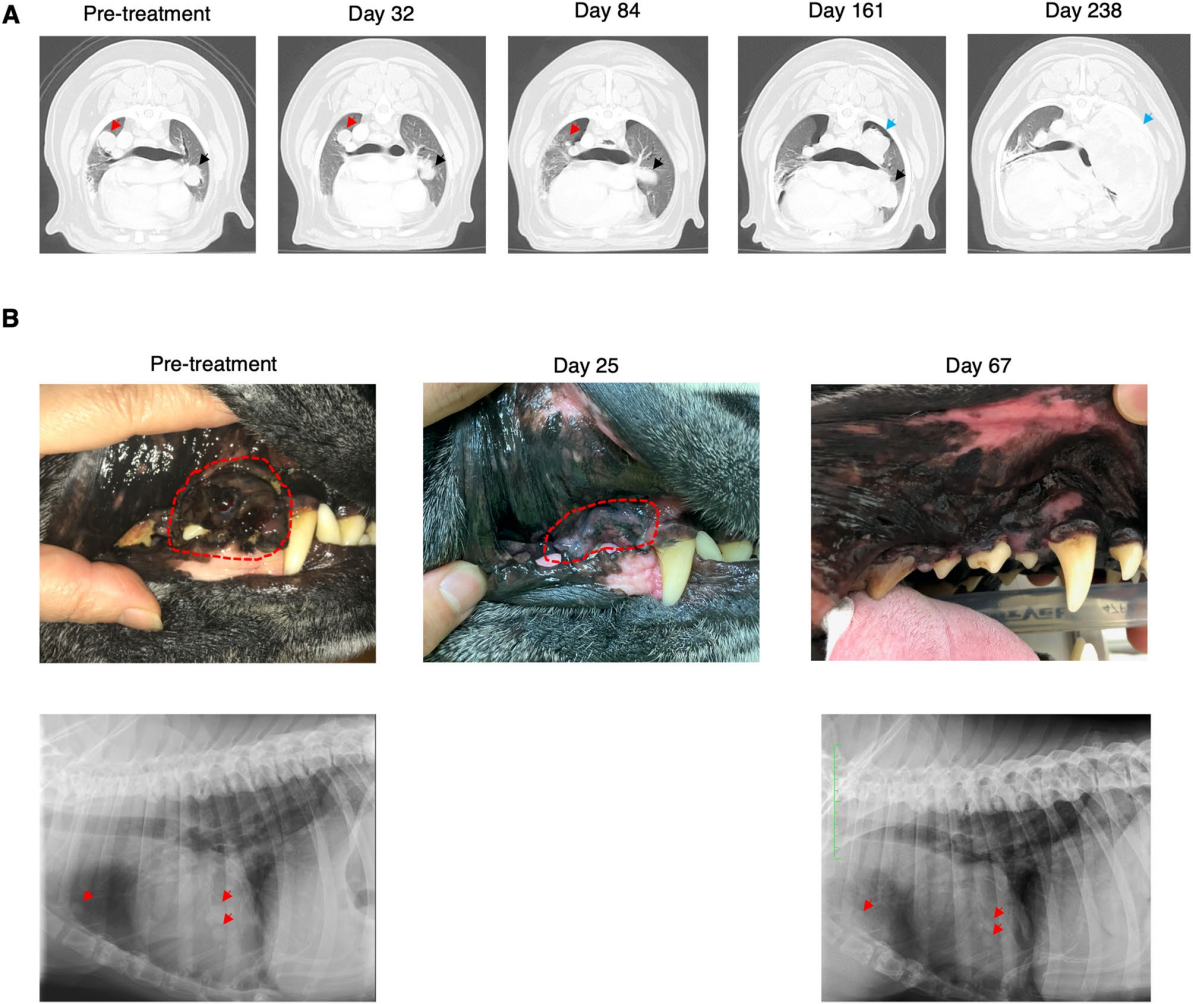
Antitumor response with anti-PD-1 antibody in two oral malignant melanoma cases. (**A**) Case 3: Representative CT scan images of lung metastasis of a Stage IV oral melanoma case treated with a total of 17 doses of ch-4F12-E6 administration. In pre-treatment, two masses were observed in this image (indicated as red or black arrows). After treatment with anti-PD-1 antibody, tumor masses were shrinking. On day 161, CT scanning revealed progressive disease due to new metastases (indicated by the blue arrow). (**B**) Case 30: A dog with oral melanoma involving lung metastasis (Stage IV) was treated with one cycle of ca-4F12-E6 administration. Tumor response rates in primary mass as target lesion and lung metastasis as non-target lesion were evaluated by caliper or X-ray, respectively. Oral primary mass completely responded to anti-PD-1 antibody on day 67, while tumor burden of lung metastasis (indicated as red arrows) was stable.

Notable case examples for Stage IV OMM are shown in Fig. 4A (Case 3), Fig. 4B (Case 30) and Supplementary Fig. S3 (Case 28). Case 3 received ch-4F12-E6 over three treatment cycles. Both Cases 30 and 28 received ca-4F12-E6 over one and four treatment cycles, respectively. Figure 4A shows a CT scan of lung metastasis in Case 3. This case had previously undergone radiation therapy without any systemic therapy. Pre-treatment CT scans revealed multiple lung metastases. The tumor masses shrunk after anti-PD-1 treatment. Interestingly, a certain mass (indicated as red arrow) regressed completely, but another one (indicated as black arrow) exhibited only weak regression. On day 161, the new lesion (indicated as blue arrow) appeared in addition to re-proliferation of the target lesion (black arrow).

In Case 30, which had primary OMM and lung metastasis with no therapeutic intervention, treatment with anti-PD-1 antibody yielded complete tumor regression of the target lesion (oral mass) and rapid resolution of symptoms such as bleeding from the tumor until day 67 (Fig. 4B). Although the tumor burden of non-target metastatic lesions (indicated as red arrow) showed only moderate regression, this case had an objective response.

Case 28, which had tumor progression for multiple lesions of lung metastasis after radiation therapy, showed progressive disease of the target lesions during treatment. However non-target lesions were significantly suppressed. The graph shown in Supplementary Fig. S3A indicates each tumor size for target lesions (tumors 1 and 2) and non-target lesions (tumors 3 and 4), over time. The size of all tumors excepting tumor 4 increased on day 72, although tumor 4 regressed completely. As an assessment criterion in the present study, this case had progressive disease. Nevertheless, we continued treatment at the owner’s request. A CT scan indicated that the size of tumor 1 had gradually increased by day 267 after four cycles of treatment (Supplementary Fig. S3B). In contrast, the growth of other tumors had diminished and no new lesions of lung metastasis were observed. These data suggested that the efficacy of anti-PD-1 treatment is variable between different tumors even in the same individual, which is similar to the mixed tumor responses identified in human studies^33,34^.

### Survival time

**Figure 5:**
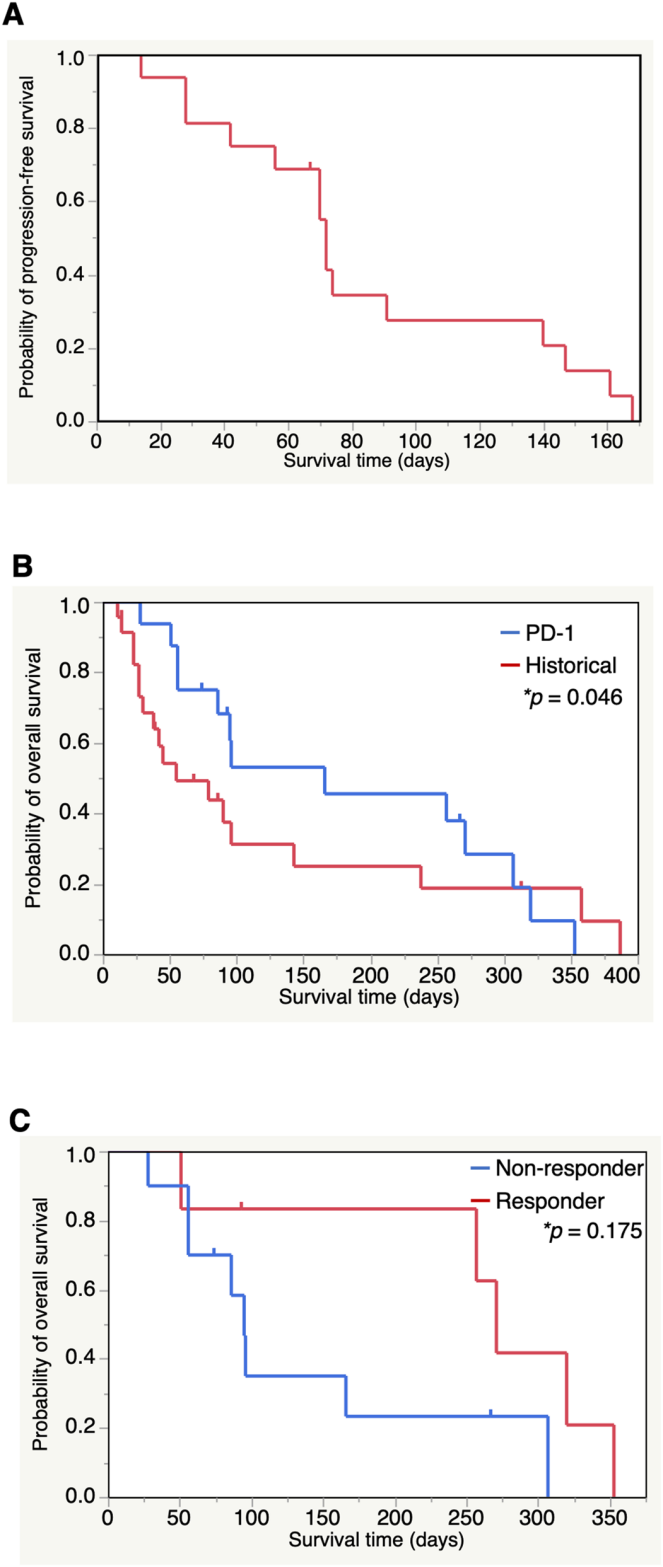
Kaplan–Meier survival curves of Stage IV oral malignant melanoma cases who received with anti-PD-1 antibody. (**A**) Kaplan–Meier curve shows the progression-free survival (PFS) of 16 dogs with Stage IV oral malignant melanoma (OMM). PFS was measured from initiation of treatment until disease progression or death, whichever occurred first. Data from one case without an event was censored at the last date of disease assessment. (**B**) Comparison of overall survival (OS) between 16 dogs with Stage IV OMM who received anti-PD-1 antibody (blue line) and historical controls including 23 dogs with Stage IV OMM who received standard therapy in our veterinary teaching hospital (red line). (**C**) Kaplan–Meier curve of OS for dogs in (**B**) that had less than 20% of maximum tumor reduction by anti-PD-1 antibody (responder group, n = 6) versus the remaining cases (non-responder group, n = 10). (**A**–**C**) Tick marks on lines indicate censored data. Statistical difference was calculated using the Wilcoxon test.

Progression-free survival time (PFS) and overall survival time (OS) in all enrolled dogs are shown in Table 1. The prolonged survival time after treatment of anti-PD-1, was further analyzed in all cases of Stage IV OMM except for Case 12. Kaplan–Meier curves for PFS and OS are shown in Fig. 5A–C. Median PFS and OS was 72 days (95% confidence interval [CI] 42–140) and 166 days (95% CI 56–307), respectively (Fig. 5A,B). To confirm whether the anti-PD-1 antibody contributed to prolonged survival times for melanoma, we compared OS for Stage IV OMM in the anti-PD-1 treatment group in the present study with the OS of a historical control group from our veterinary teaching hospital. The 23 cases of Stage IV OMM used as historical controls had received standard therapies (surgery, radiation, and chemotherapy) and/or DNA vaccine encoding human tyrosinase and oncolytic virotherapy (Supplementary Table S1). Survival time in the historical controls was defined as the time period from the confirmation of lung metastasis to death or last follow-up. The median OS in historical controls (comprised of 18 events and 5 censored) was 55 days (95% CI 27–143). The OSs of the two groups were shown to be significantly different using the Wilcoxon test (p = 0.046) (Fig. 5B).

When Stage IV OMM cases that received the anti-PD-1 antibody were separated into two groups: responder (n = 6) and non-responder (n = 10). The median OS by Kaplan–Meier analysis was 271 days (95% CI 51–353) and 95 days (95% CI 28–307), respectively (p = 0.175) (Fig. 5C). Based on the results of clinical assessment, we concluded that the anti-PD-1 antibody is a potentially effective treatment for dogs with advanced OMM.

### Detection of anti-drug antibody and relation to the concentration of administered antibody

Our engineered antibodies, ch-4F12-E6 and ca-4F12-E6, reduced the immunogenicity and the risk of producing anti-drug antibodies. However, the repeated administration of these antibodies might trigger the dog’s immune response, leading to production of anti-drug antibodies, which may neutralize or reduce the antitumor response, and increase adverse events^35,36^. Methods for detection of anti-drug antibodies have been investigated by many researchers, but a consensus has yet to be reached regarding the most appropriate method. We ran preliminary tests in-house using ELISA to measure levels of anti-drug antibodies. Serum samples from 26 of the 30 cases taken on the date of visit to the clinic were used (Table 1). In two cases (Case 24 and Case 27) samples taken after treatment showed elevated absorbance values compare to their baseline samples (Supplementary Fig. S4). The results of Case 25 are shown as representative of all other cases which showed no increase in absorbances.

To confirm the association between anti-drug antibodies and administered antibodies, we measured the blood concentrations of those antibodies by ELISA using canine PD-1 protein fused human IgG Fc region. Standard curves of anti-PD-1 antibody were used for calculating the concentration of each sample. Serum concentration–time profiles of anti-PD-1 antibody are shown in Supplementary Fig. S5. Blood concentrations of anti-PD-1 antibody remained constant for at least two weeks after each administration.

Notably, the presence of anti-drug antibodies in Cases 24 and 27 was not accompanied by low blood levels of administered antibodies. Hence, the role of anti-drug antibodies in dogs remains unknown. Nevertheless, these results revealed that our engineered antibodies have lower immunogenicity in dogs.

## Discussion

In the present study, we developed therapeutic monoclonal antibodies, by chimerization and PETization, that had lower immunogenicity and recognized canine PD-1 protein. Both antibodies had binding affinity to PD-1 and an inhibitory effect on PD-1/PD-L1 interaction to the same extent as the original rodent antibody^19^. During the IFN-γ releasing assay, caninized ca-4F12-E6 antibody blocked the PD-1-mediated suppression of T-cell activation by the PD-L1 protein. However, the levels of IFN-γ production varied between donors, as demonstrated in previous studies^16,19,20^. We hypothesized that the variation of IFN-γ production might have arisen from dog’s individual characteristics including age, stress, the degree of T-cell activation, and the expression of PD-1 protein. Nevertheless, we found evidence for the functionality of our established chimeric and caninized anti-PD-1 antibodies.

In this clinical study, PD-1 blockade exhibited safety and antitumor activity in cases of OMM with lung metastasis. Objective partial responses of anti-PD-1 treatment occurred in four of 15 (26.7%) measurable Stage IV OMM cases. Although the objective response rate was low in comparison with human advanced melanoma^37^, this result suggests the therapeutic potential of PD-1 blockade in canine melanoma. Due to the lack of previous studies describing the clinical efficacy of anti-PD-1 antibody in dogs, we compared survival times of experimental cases with historical controls from our veterinary teaching hospital. One previous study reported the development of chimeric monoclonal antibodies against canine PD-L1 protein and performed a clinical trial of these in canine OMM cases at various stages of disease^20^. The study used historical cases of Stage IV OMM from their hospital as controls, and the median OS was 54 days, which is similar to the median OS of 55 days obtained for our historical controls. Although historical comparison of median OS should be viewed with caution, it indicates that treatment with the anti-PD-1 antibody could extend survival in dogs with advanced OMM.

In addition to OMM cases, MGT, SCC, skin melanoma, renal carcinoma, lymphoma, sebaceous carcinoma, and lung adenocarcinoma cases were enrolled in this study. PD-1 and PD-L1 blockade therapies are used in a wide range of human cancers. SCC, skin melanoma, renal carcinoma, and non-small cell lung cancer, in particular are good targets for these therapies^9,10^. Since the number of cases in this study was very small, further clinical studies of various types of canine tumors are recommended.

Recently, anti-PD-1/PD-L1 antibodies combined with standard chemotherapy or radiation have been intensively conducted in human’s clinical trial because those therapies were reported to enhance the antitumor immune response^38,39^. Twenty seven out of 30 cases had received prior therapies including surgery, radiation, systemic chemotherapy, and other immunotherapy before initiation of the present study, and were judged as refractory to those prier therapies when anti-PD-1 antibody therapy started. However, the present data of antitumor response might be interpreted with some cautious because the prior therapies may effect on the response to anti-PD-1 antibody therapy. On the other hand, Case 30 with no therapeutic intervention indicated partial response after anti-PD-1 treatment. To determine the sole effect of anti-PD-1 antibody in canine tumors, clinical studies of treatment-naïve dogs with tumors are required.

Treatment with the anti-PD-1 antibody was relatively safe and well tolerated although two cases had adverse events greater than Grade 3. The observed adverse events were similar to those of humans, except that endocrine, skin, and vascular disorders were not observed. Among adverse events of special interest, the most severe adverse event in the present study was an immune-related pneumonitis of Grade 5 in Case 10, which occurred after three doses of anti-PD-1 antibody. A previous human phase 1 clinical trial reported three deaths occurring due to pneumonitis with no clear relationships to dose levels, tumor type, or the number of doses received^31^. A systematic review and meta-analysis of malignant melanoma with immune-related adverse events revealed that the incidences of pneumonitis (including all grades) after nivolumab was 1.4% and after pembrolizumab was 2.0%^40^. In addition, hepatitis is a common immune-related adverse event reported after treatment of immune checkpoint inhibitors. The incidence of hepatitis of all grades associated with nivolumab is 3% in melanoma patients^40^. In the present study, Case 1 showed severe hepatic injury after eleven doses of anti-PD-1 antibody followed by the restoration of levels of liver enzymes after administration (guided by human studies) of a glucocorticoid and an immunosuppressive drug^41,42^. We were, however, unable to establish a clear reason for the severe adverse events associated with anti-PD-1 antibody treatment. Even in human studies, the risk of onset and management of immune-related adverse events present challenges. Thus, the accumulation of knowledge resulting from studies involving anti-PD-1 antibody treatment in dogs is useful not only in assisting veterinarians with the management of canine cases, but also for informing future human research studies. Overall, the safety profiles of our established antibodies suggest that anti-PD-1 treatment may safely be delivered to dogs with tumors with minimal supportive care.

PD-1 blockade therapy has effectively prolonged survival time in some, but not all human cancer patients. Hence, the establishment of a valid biomarker for treatment–response has become an urgent priority. So far, various predictive markers have been reported including PD-L1 expression, mutation burden, and lymphocyte infiltration^43–46^. In the present study, subgroup analysis for median OS in Stage IV oral malignant cases indicated that treatment–response cases tended to prolong survival when compared to non-response cases, but the difference did not reach statistical significance. Hence, we reasoned that, as was the case in humans, investigation of biomarkers is required in future clinical studies in dogs. The expression of PD-L1 is one of the major biomarkers in human clinical trials because PD-L1 binds to the PD-1 protein on lymphocytes, which is a negatively regulated immune response. However, in the present study, the expression status of PD-L1 on tumor tissues could not evaluated in the enrolled dogs due to lack of an available antibody for paraffin-embedded immunohistochemical staining. Therefore, to analyze the correlation of PD-L1 expression and prognosis in dogs receiving anti-PD-1 antibody treatment, we are attempting to develop a mouse monoclonal antibody against canine PD-L1.

Anti-drug antibodies were assessed to determine the immunogenicity of our administered antibodies that could potentially lead to an increase in adverse events or loss of antitumor efficacy. Multiple methods for the detection of anti-drug antibodies have been established in humans, but not yet dogs. We first prepared the HRP-conjugated anti-dog IgG secondary antibody that could recognize anti-drug antibodies, but not administered antibodies, in sera. Canine IgG was categorized into four subclasses (type A, type B, type C, and type D), which corresponded to human IgG analogs^47,48^. Our established antibodies comprised the type A subclass and therefore a drawback of this assay is that secondary antibodies cannot recognize IgG type A isotypes of anti-drug antibodies. Despite not being able to cover all subclasses of anti-drug antibodies in this study, we detected two anti-drug antibody-positive cases out of 26 cases by conventional sandwich ELISA. Recent human studies use a bridging ELISA technique in which a biotinylated administered antibody is developed to detect the anti-drug antibody^49–52^. A limitation to this is that the bridging ELISA is unable to detect anti-drug antibodies complexed to the administered antibody in sera^51,53^. In order to overcome these limitations, we must investigate and validate a detection method for anti-drug antibodies in canine samples.

This present study and the previous study using anti-PD-L1 antibody^20^ first described the clinical efficacy of immune checkpoint inhibitors in the veterinary field, indicating the significance of the PD-1/PD-L1 interaction in canine OMM. However, both studies included a few treatment-naïve cases and the limited types of tumors. To expand these observations, we must attempt investigating the efficacy of PD-1 or PD-L1 antibodies in various types of canine tumors, and the predictive biomarkers to optimize case/tumor selection, minimize risk of adverse events, and prognosis. Since the differences in the efficacy or safety between anti-PD-1 and anti-PD-L1 immunotherapies have not been undefined even for human, it might be difficult to perform clinical studies to prove the superiority of ours and Maekawa’s group’s one^20^ with a sufficient number of tumor cases in veterinary field. However, one can predict that the required dosage of anti-PD-1 antibody may be lower than anti PD-L1 antibody, which binds to tumor cells and/or immune cells expressing PD-L1 protein. This might lead to give the difference of the cost of antibody.

In conclusion, we developed chimeric and caninized anti-PD-1 monoclonal antibodies and confirmed the bioactivity of those antibodies compared to the original rat antibody. In addition, a pilot clinical study provided the first evidence in veterinary medicine that anti-PD-1 monoclonal antibodies are relatively safe, well tolerated, and effective in treating some dogs with spontaneous tumors. However, there were some limitations. First, a dose-escalation trial of anti-PD-1 antibody was not conducted. Second, the number of cases with each tumor type was small. Third, more optimal disease control groups from other facilities would be useful. Nonetheless, the present study supports further investigation into anti-PD-1 antibody therapy for canine OMM and other types of tumors, and we anticipate that solid biomarkers including PD-L1 expression and other emerging candidates will be established.

## Materials and methods

### Ethics statement of experimental animals and clinical cases

The use of PBMCs isolated from specific pathogen-free dogs was approved by the Institutional Animal Care and Use Committee of Yamaguchi University (Permit Number: 188). The present clinical study using owners’ dogs was approved by the Ethics Review Board of the Joint Faculty of Veterinary Medicine of Yamaguchi University. Also, their owners all signed informed-consent forms. All experiments were conducted in accordance with relevant guidelines and regulations of the United Graduate School of Veterinary Medicine, Yamaguchi University.

### Study design

This clinical study was conducted at Yamaguchi University Animal Medical Center (YUAMEC). The primary objectives of this trial were to determine the safety profile and tolerability of anti-canine PD-1 monoclonal antibody therapy in dogs with advanced cancers. The secondary objectives included assessing antitumor effects and OS time in enrolled dogs. Additionally, the concentration of anti-canine PD-1 administered antibody and anti-drug antibody (anti-idiotypic antibody) to the administered antibody in serum were measured. All enrolled dogs received treatment between April 21, 2017 and April 8, 2020. The eligibility criteria included a confirmed tumor diagnosis by cytology or pathology, a tumor mass that allowed evaluation, and a life expectancy of at least one month with a standard therapy. Cases receiving concurrent treatments such as systemic chemotherapies were excluded. However, the use of concurrent continuous treatments with tyrosine kinase inhibitor toceranib, metronomic therapy using cyclophosphamide, or non-steroidal drugs was accepted if cases had already received those treatments with no apparent antitumor effects.

Either anti-canine PD-1 chimeric antibody, ch-4F12-E6, or caninized antibody, ca-4F12-E6, was administered at 3 mg/kg as an intravenous infusion for 1 h every two weeks of each 10-week treatment cycle. From April 21, 2017 until February 1, 2019, ch-4F12-E6 was administered and, from February 6, 2019 until April 8, 2020, ca-4F12-E6 was used. Treatment was discontinued if one of the following occurred: owner made the decision to withdraw, there was an unacceptable adverse event, disease progression or changes in the dog’s condition prevented further treatment in the opinion of the veterinarian. In clinically stable cases with owner’s permission, treatment was continued beyond apparent initial disease progression until further progression was confirmed.

Safety assessment was performed in all enrolled dogs from initiation to trial termination. Adverse events were graded according to the Veterinary Cooperative Oncology Group—Common Terminology Criteria for Adverse Events (VCOG-CTCAE) v1.1 scale^54^.

### Patients

Eligible cases had documented treatment resistant, refractory, or advanced staging and comprised 21 OMM (4 cases with stage III and 17 cases with stage IV), 2 skin malignant melanoma, 2 MGT (one with simultaneous sebaceous carcinoma), 2 SCC, 1 renal carcinoma, 1 lymphoma, and 1 lung adenocarcinoma. All cases were restaged by radiography (X-ray, CT scan, or ultrasound) before the initiation of the present study. A summary of baseline characteristics, treatment course, anti-drug antibody, and survival time in all enrolled dogs is shown in Table 1.

### Procedure

To determine the nucleotide sequence of the variable region of the light and heavy chains of 4F12-E6 rat antibody, we performed 5′-rapid amplification of cDNA ends (RACE), as described in previous our report. The variable region of the 4F12-E6 heavy and light chains were ligated to the constant regions of canine IgG subclass A^47^ by overlapping PCR and ligated into the pCAGGS-MCS vector.

To obtain the rat-dog chimeric antibody (ch-4F12-E6), expression plasmids for the heavy and light chains were transfected into 293 cells using the Expi293 expression system (Thermo Fisher Scientific, Waltham, MA, USA). The collected supernatant was purified using a HiTrap Protein A HP column (GE Healthcare Life Sciences, Pittsburgh, PA, USA) and desalted using a PD10 column (GE Healthcare Life Sciences).

To further decrease the rat-derived sequence, conversion of the heavy and light chain variable domain framework sequence from rat to dog, known as PETization, was conducted^55^. By this process, the rat framework sequence of 4F12-E6 was completely caninized by our collaborator, Nexvet (Melbourne, Australia). During production of the caninized anti-canine PD-1 antibody ca-4F12-E6, the expression vectors were stably transfected to CHO-S cells. The culture supernatant was purified as described above.

To obtain the isotype control antibody for in vitro assay, the expression vectors coding dog IgG subclass A (Dog IgG-A) with relevant CDRs to canine sequence of heavy and light chains were prepared in a similar method to ch-4F12-E6.

### Flow cytometry

To assess the binding characteristics of ch-4F12-E6 and ca-4F12-E6 to canine PD-1 protein, we used a rat kidney cell line NRK which stably expressed canine PD-1 (NRK/cPD-1) established in our previous study^19^. Harvested cells were stained with primary antibodies (40 μg/ml) including rat IgG2a isotype control (BioLegend, San Diego, CA, USA), 4F12-E6, ch-4F12-E6, and ca-4F12-E6, followed by incubation with Dylight 649-conjugated goat anti-rat IgG (BioLegend) or AlexaFluor 647-conjugated rabbit anti-dog IgG (Jackson ImmunoResearch, West Grove, PA, USA). The cells were stained with propidium iodide to gate out dead cells. To discriminate positive from negative staining cells, we used either a no-staining control or a secondary antibody-only control.

To determine the blocking ability for PD-1/PD-L1 binding, we first prepared recombinant canine PD-L1 protein fused to human IgG Fc (cPD-L1-Ig) as described in Supplementary methods. NRK/cPD-1 was added at 2 × 10^5^ cells per well in a 96-well U bottomed microwell plate and pre-incubated with dose-titrated ch-4F12-E6 and ca-4F12-E6 (0, 0.16, 0.63, 2.5, 10, 40 μg/ml) for 30 min at 4 °C. After washing, human Ig and cPD-L1-Ig proteins (10 μg/ml) were added and incubated for 30 min at 4 °C. cPD-L1-Ig binding was detected using PE-conjugated goat anti-human FC gamma fragment specific antibody (Jackson ImmunoResearch).

All stained samples were analyzed by BD Accurri C6 flow cytometer (BD Biosciences, San Joes, CA, USA). All given data were analyzed using Flowjo software v10.5.3 (Flowjo, Ashland, OR, USA).

### PBMC isolation, T-cell stimulation, and interferon (IFN)-γ releasing assay

PBMCs from three specific pathogen-free dogs (maintained as blood donors at YUAMEC) were isolated by density gradient centrifugation, using Lymphoprep (Axis-Shield, Oslo, Norway). For the interferon (IFN)-γ production assay, isolated PBMCs were seeded in duplicate at 2 × 10^5^ cells per well into a 96-well U bottomed plate and stimulated with 5 μg/ml of concanavalin A (ConA). Simultaneously, 10 μg/ml of ca-4F12-E6 or dog IgG-A control were added and incubated at 37 °C in a CO_2_ incubator. After 24 h of incubation, 10 μg/ml of cPD-L1-Ig and human Ig protein were added, and samples were incubated for 48 h. Supernatant from each sample was collected and the amount of IFN-γ was measured using a DuoSet ELISA Development System for canine IFN-γ (R&D systems, Minneapolis, MN, USA).

### Clinical assessment

All enrolled dogs were restaged for disease progression either by caliper measurement or radiography (X-ray and CT scan) and comparisons were drawn between baseline and termination of each treatment cycle. All baseline evaluations were performed as close as possible to treatment initiation and no longer than 1 week prior to the start of treatment. According to the response evaluation criteria for solid tumors in dogs (cRECIST v1.0)^56^ the minimum size of a target lesion is defined as a tumor diameter of at least 10 mm. Tumors smaller than this 10-mm limit were classified as non-target lesions. Most of the enrolled dogs had metastatic tumors smaller than 10 mm in diameter, which were derived from target lesions defined by cRECIST v1.0. Therefore, response evaluation in the present study was defined according to cRECIST v1.0 as follows: (1) primary mass was measured if the tumor had been unaffected by prior therapy (e.g., surgery, radiation) and was accurately measurable by caliper or CT scan; (2) metastatic mass was measured by X-ray and CT scan in cases where there the primary site was not measurable, (3) metastatic masses were considered to be measurable lesions even if the longest diameter was less than 10 mm, (4) when there were multiple target lesions at baseline, a maximum of five target lesions, with a maximum of two lesions in the same organ, were chosen. We chose two target lesions per an organ in descending order of size.

The criteria of tumor response were defined as follows: progressive disease (PD) constituted either at least a 20% increase in the sum of the diameters of target lesions from baseline or the appearance of new metastatic lesions; stable disease (SD) was defined as less than 20% increase or 30% reduction in the sum of the diameters; PR comprised at least 30% reduction in the sum of the diameters; and complete response required the disappearance of all target lesions.

Progression-free survival (PFS) was defined as the length of time from the initial treatment until PD or death at the end of study, and overall survival (OS) as time from the initial treatment until death of cases at the end of this study. In Fig. 5C, cases were categorized into the responder group if the percentage of maximum tumor reduction was less than 20%. Historical controls included 23 OMM cases (Stage IV) treated with standard regimens, human tyrosinase DNA vaccine, and oncolytic virotherapy^57^ at YUAMEC from March 1, 2016 until February 4, 2020. The purpose of this selection was to allow for a comparison of the results of anti-PD-1 therapy with those of conventional therapies in advanced OMM in our veterinary hospital.

### Statistical analysis

The mean values in the ELISA data set were calculated from the mean value of two technical independent experiments and shown as bar graphs. The dots on each bar indicate the raw values of two independent technical experiments. JMP 14 software (SAS Institute Japan, Tokyo, Japan) was used in the present study and survival curves were designed by Kaplan–Meier method and compared using the Wilcoxon test. Differences were considered statistically significant if p-values were less than 0.05.

## Supplementary information

Supplementary Figures.

Supplementary Information 1.

Supplementary Table 1.
