# Supplementary material for: A pilot clinical study of the therapeutic antibody against canine PD-1 for advanced spontaneous cancers in dogs
Source: Sci Rep. 2020 Oct 27;10:18311. doi: 10.1038/s41598-020-75533-4 (PMC7591904; doi:10.1038/s41598-020-75533-4)

Supplementary Figure S1

A

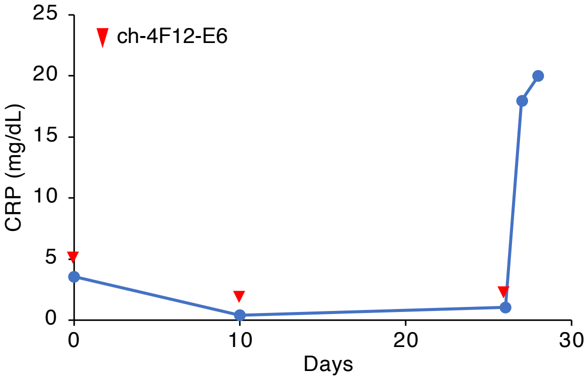

B

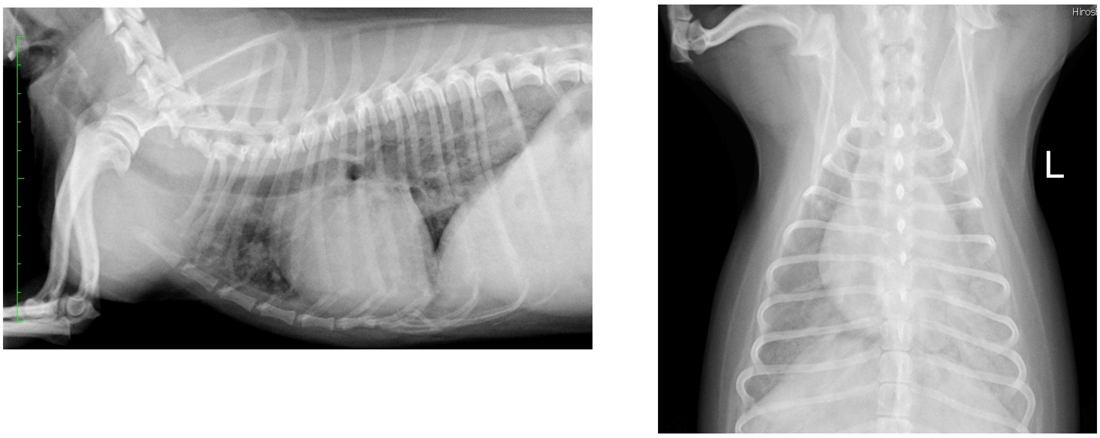

Supplementary Figure S2

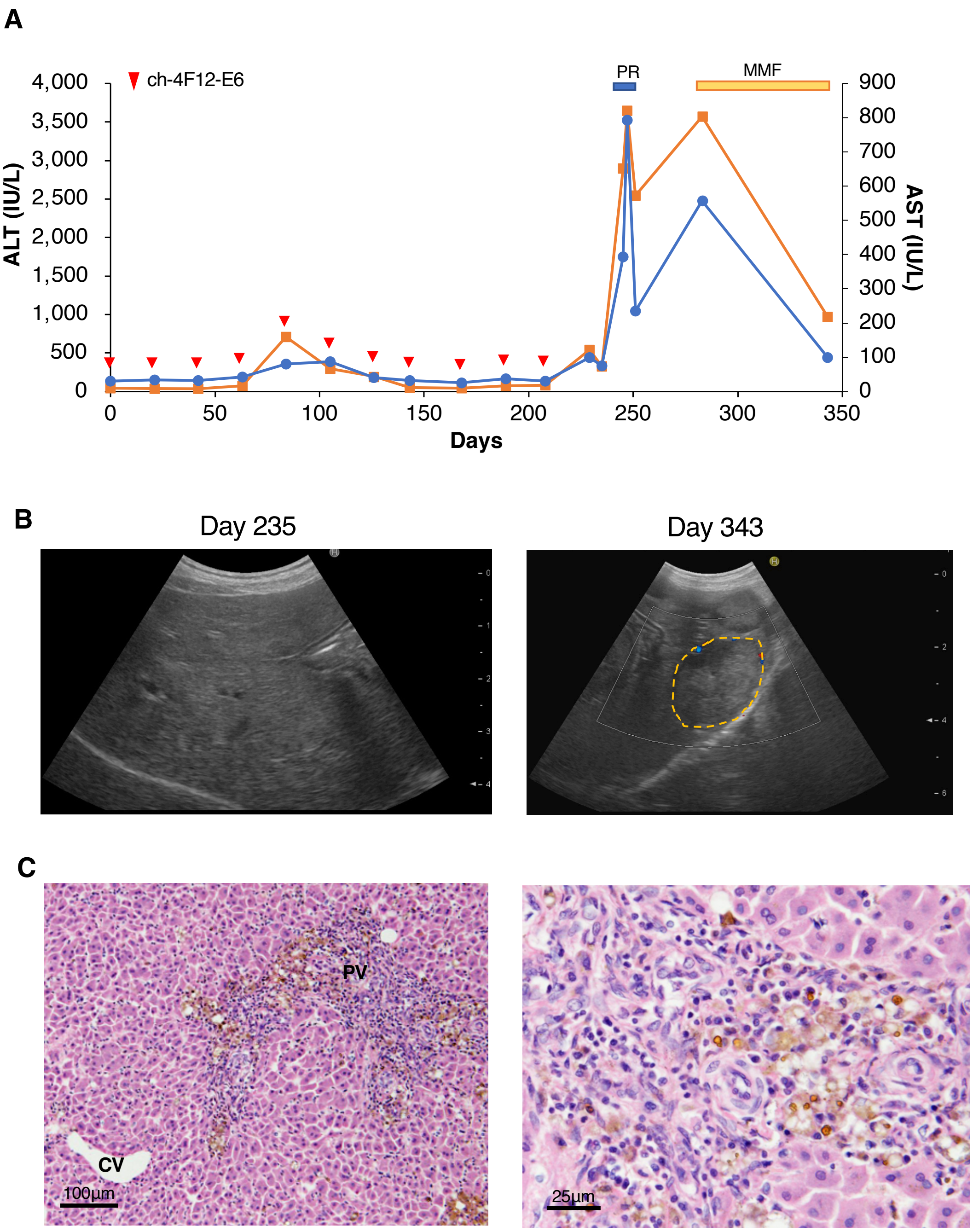

Supplementary Figure S3

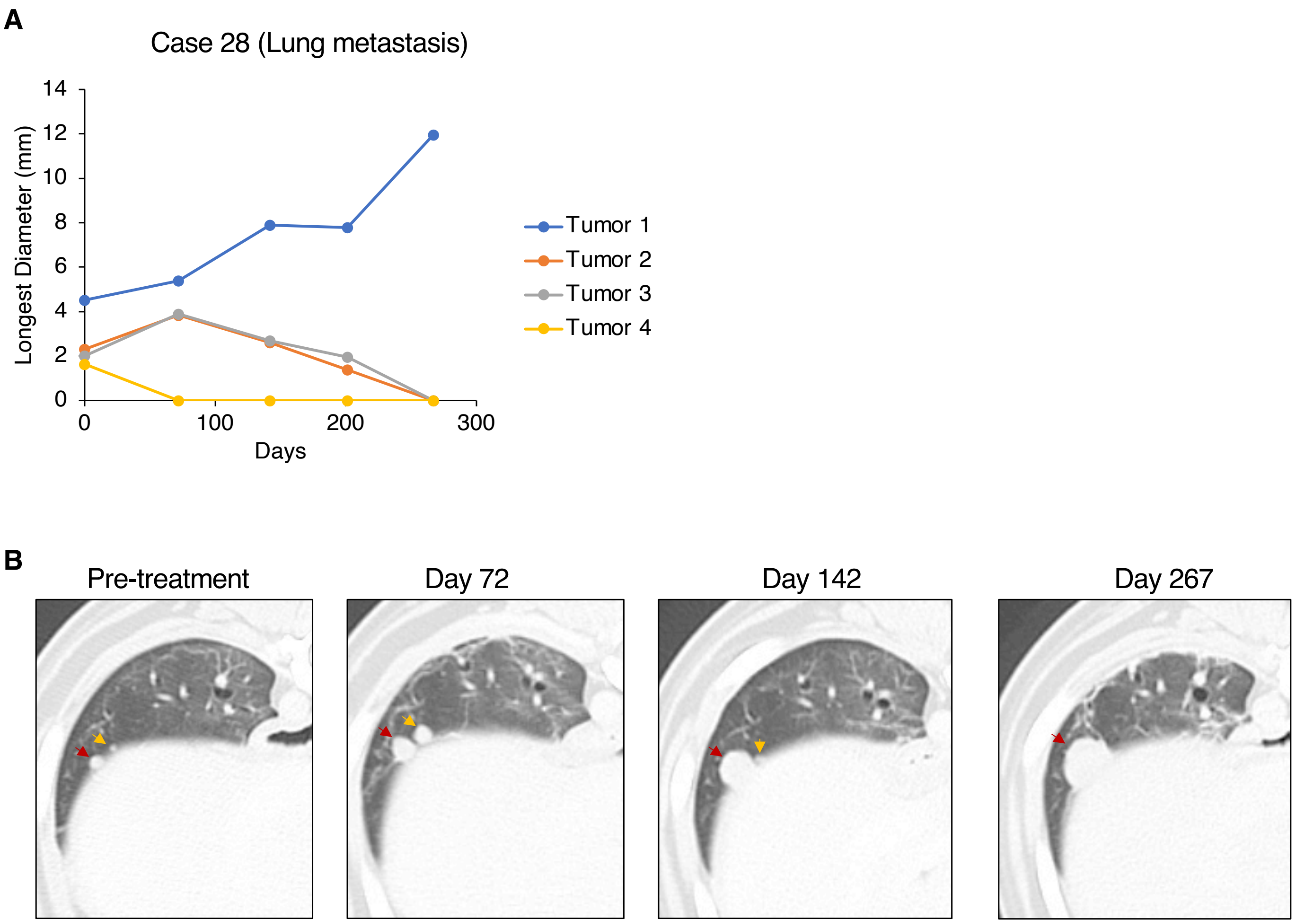

Supplementary Figure S4

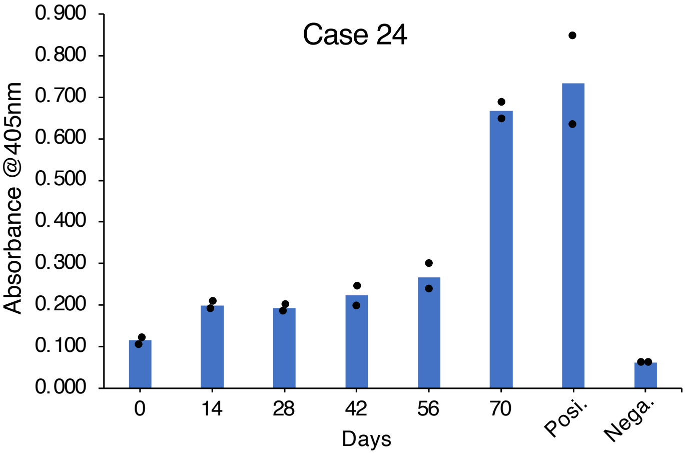

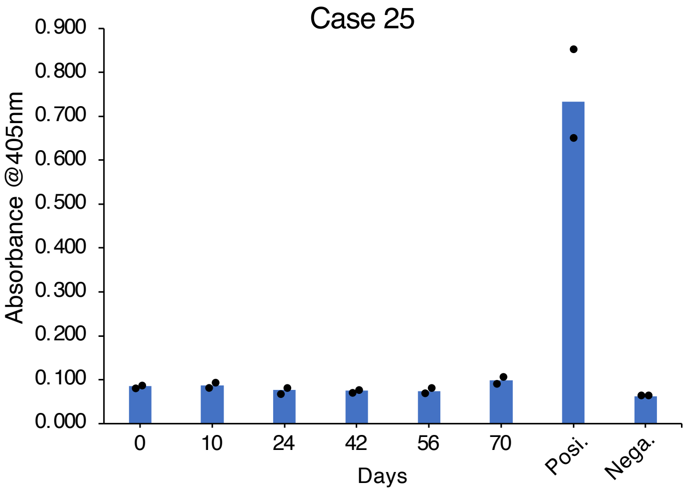

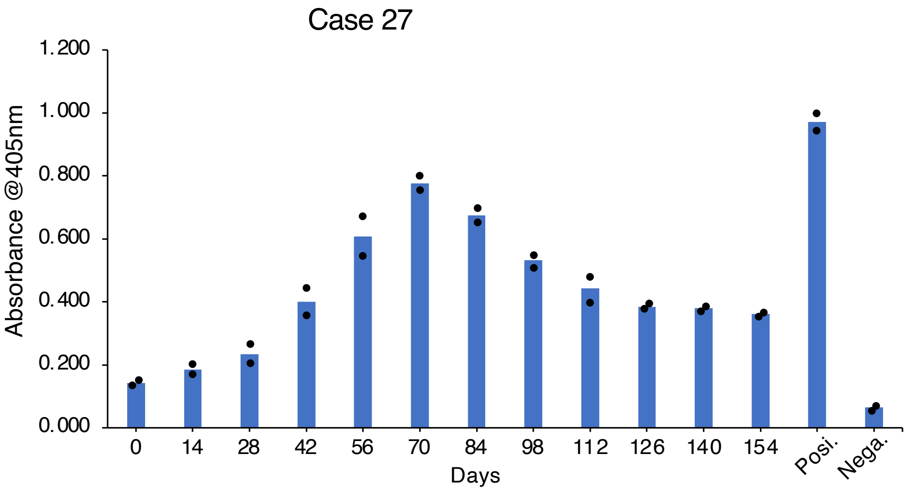

Supplementary Figure S5

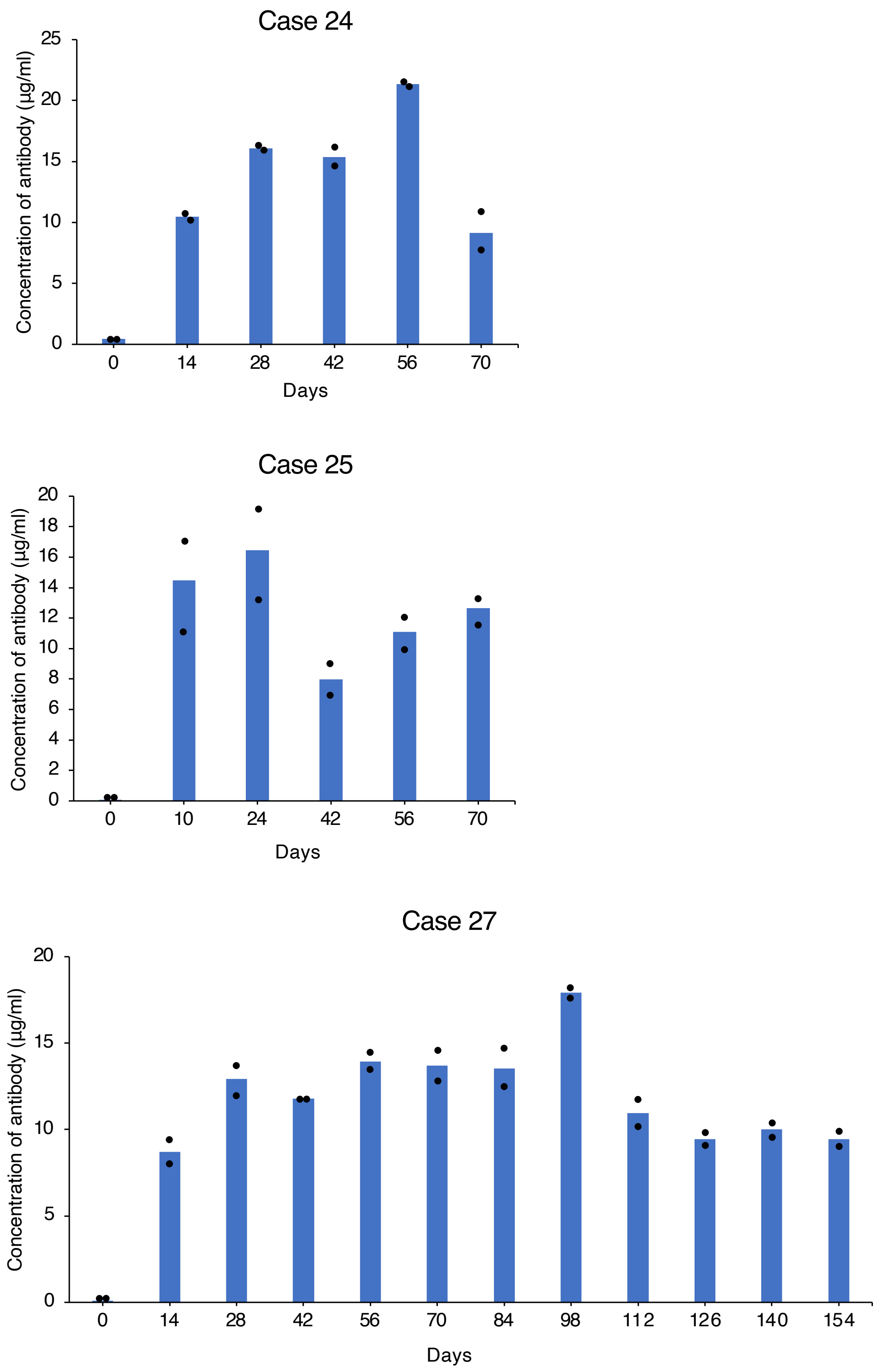

Supplement: Supplementary file 1 — Supplementary Figures. [file 41598_2020_75533_MOESM1_ESM.pdf]
