# Supplementary material for: A pilot clinical study of the therapeutic antibody against canine PD-1 for advanced spontaneous cancers in dogs
Source: Sci Rep. 2020 Oct 27;10:18311. doi: 10.1038/s41598-020-75533-4 (PMC7591904; doi:10.1038/s41598-020-75533-4)
Supplement: Supplementary file 2 — Supplementary Information 1. [file 41598_2020_75533_MOESM2_ESM.docx]

**A pilot clinical study of the therapeutic antibody against canine PD-1 for advanced spontaneous cancers in dogs**

Masaya Igase, Yuki Nemoto, Kazuhito Itamoto, Kenji Tani, Munekazu Nakaichi, Masashi Sakurai, Yusuke Sakai, Shunsuke Noguchi, Masahiro Kato, Toshihiro Tsukui, Takuya Mizuno^*^

^*^Corresponding Author

**Supplementary Methods**

**Flow cytometry**

Constructs pFUSE-cPD-L1-hIg#8 and empty vector pFUSE-hIgG2-FC2 were described in our previous study^19^. The expression vector pFUSE-cPD-L1-hIg#8 was transfected into ExpiCHO-S cells, followed by the production of cPD-L1-Ig protein using the ExpiCHO Expression System Kit (Thermo Fisher Scientific) according to the manufacturer’s protocol. The empty vector pFUSE-hIgG2-FC2 was transfected into HEK293T cells, as a control, using Polyethylenimine MAX (Polysciences, Warrington, UK). Supernatant from each transfected culture was purified by rProtein A agarose (GE Healthcare Life Scinences). The soluble cPD-L1-Ig and human Ig control were desalted via dialysis. Their purity was confirmed by SDS-PAGE and western blotting. These recombinant proteins were used in the blocking assay.

**Detection of anti-drug antibody and administered antibody**

In order to detect the production of anti-idiotypic antibodies (anti-drug antibodies) against the administrated antibody, we collected sera from all enrolled dogs at various time points and kept the samples at -80 °C. These samples were analyzed by Enzyme-Linked ImmunoSorbent Assay (ELISA).

To detect anti-drug antibody, 1 μg/ml of ch-4F12-E6 or ca-4F12-E6 was coated on each well of a Maxisorp 96-well microplate (Thermo Fisher Scientific). The plate was incubated at 4°C overnight and washed three times with a wash buffer of phosphate-buffered saline (PBS) containing 0.05% Tween-20. Blocking buffer of 3% bovine serum albumin in PBS was added and samples incubated for 1 hour at 37°C. After washing, each serum was diluted 1/100 in, blocking buffer was added and samples incubated as mentioned above. The plate was washed five times and incubated with HRP-conjugated mouse anti-dog IgG (diluted; 1:5000, Invitrogen, Carlsbad, CA, USA), which was non-binding to dog IgG type A and administered antibodies. To obtain the serum showing positivity against ch-4F12-E6, we immunized two healthy SPF rabbits (Oriental Yeast, Tokyo, Japan) intramuscularly with ch-4F12-E6 and harvested sera from the rabbits one month later. The resulting rabbit anti-4F12-E6 polyclonal antibody was used as a positive control. Each sample was assayed in duplicate. A positive result for anti-drug antibody referred to the absolute value of absorbance compared to the baseline absorbance of the pre-treatment sample (day 0) or the absorbance of positive control in each case.

To measure the concentrations of administered antibodies, 1 μg/ml of recombinant canine PD-1 fused to human IgG Fc (cPD-1-Ig), as described previously^19^, was coated on a Polysorp 96-well microplate (Thermo Fisher Scientific) and incubated at 4°C overnight. After washing, each serum was diluted 1/1000 in h-Block-e blocking reagent (Beacle, Kyoto, Japan) which is used as the blocking buffer and added into each well. The standard curve was generated using each of the administered antbodies, ch-4F12-E6 and ca-4F12-E6. The plate was washed and incubated with HRP-conjugated goat anti-dog IgG (diluted 1:5000, SeraCare Life Sciences, Milford, MA, USA).

To measure fluorescence values, a substrate ABTS Microwell Peroxidase Substrate kit (SeraCare Life Sciences) was added and incubated for 5–10 min at 37°C. The plate was measured at 405 nm by an ARVO X4 fluorometer (PerkinElmer, Waltham, MA, USA).

**Supplementary Figure legends**

**Figure S1. A treatment-related pneumonitis of grade 5 in Case 10**

(A) The graph shows chronological changes in the levels of C-reactive protein (CRP) and a summary of ch-4F12-E6 administration (indicated by red arrows). After the third administration of ch-4F12-E6, a dramatic increase in CRP was observed. (B) Thoracic X-ray on day 28 revealed severe pneumonitis.

**Figure S2. A treatment-related adverse event of liver damage in Case 1**

Case 1 had oral malignant melanoma (Stage IV) and received a total of 11 doses of ch-4F12-E6. (A) The graph shows chronological changes in liver enzymes and a summary of treatments. After the eleventh treatment, alanine aminotransferase (ALT, indicated as an orange line) and aspartate aminotransferase (AST, indicated as a blue line) were significantly increased. In addition to discontinuing anti-PD-1 antibody treatment, prednisolone treatment was initiated (PR, 1-2 mg/kg) from day 245 until day 251. Due to a side effect of the glucocorticoid, it was replaced by the immunosuppressive drug mycophenolate mofetil (MMF, 11 mg/kg) from day 283. (B) Representative image of ultrasound showing hepatic lesion on day 235 and day 343. (C) Microscopic images of liver in hematoxylin and eosin (HE) staining from autopsy on day 353. The image of the high-power field shows the portal vein area. The central vein area and portal vein area show CV and PV, respectively. The scale bars of the low- and high-power fields indicate 100 μm and 25 μm, respectively.

**Figure S3. Detailed description of tumor response to anti-PD-1 antibody in Case 28**

(A) Measurement of the longest diameter of each lung metastasis in Case 28. Tumor 1 and tumor 2 were registered as target tumors for assessment, while tumor 3 and tumor 4 were non-target lesions. (B) The images of CT scan show tumor masses pre-treatment, and on days 72, 142, and 267. Red and yellow arrows indicate the masses of tumor 1 and tumor 3, respectively.

**Figure S4. Detection of serum anti-drug antibody**

Serum anti-drug antibody over time was measured by ELISA and mean absolute absorbance values from duplicates are displayed. Among the 26 enrolled cases, only Cases 24 and 27 were positive for anti-drug antibodies. The results of Case 25 are shown as representative of all other cases which showed no increment. Rabbit anti-4F12-E6 polyclonal antibody was used as positive control. Serum collected from a healthy beagle was used as a negative control. Mean values of duplicates are displayed as a bar graph.

**Figure S5. Measurement of the blood concentrations of the administered antibody**

Serum concentrations of the administered antibody over time relative to anti-drug antibody were measured by ELISA. Standard curves of anti-PD-1 antibody were used for calculating the concentration of each sample. Mean values of duplicates are displayed as a bar graph.
